# Exercise-induced myocardial T1 increase and right ventricular dysfunction in recreational cyclists: a CMR study

**DOI:** 10.1007/s00421-023-05259-4

**Published:** 2023-07-22

**Authors:** Olivier Ghekiere, Lieven Herbots, Benjamin Peters, Baptiste Vande Berg, Tom Dresselaers, Wouter Franssen, Bernard Padovani, Dorothee Ducreux, Emile Ferrari, Alain Nchimi, Sophie Demanez, Ruben De Bosscher, Rik Willems, Hein Heidbuchel, Andre La Gerche, Guido Claessen, Jan Bogaert, Bert O. Eijnde

**Affiliations:** 1 Faculty of Medicine and Life Sciences/LCRC (-MHU), Hasselt University, Agoralaan, 3590 Diepenbeek, Belgium; 2 Department of Radiology and Department of Jessa & Science, Jessa Hospital, Stadsomvaart 11, 3500 Hasselt, Belgium; 3 Heart Centre, Jessa Hospital, Stadsomvaart 11, 3500 Hasselt, Belgium; 4 Department of Radiology, University Hospitals Leuven, Leuven, Belgium; 5 SMRC Sports Medical Research Center, BIOMED Biomedical Research Institute, Faculty of Medicine and Life Sciences, Hasselt University, Diepenbeek, Belgium; 6 REVAL-Rehabilitation Research Center, Faculty of Rehabilitation Sciences, Hasselt University, Diepenbeek, Belgium; 7 Department of Nutrition and Movement Sciences; NUTRIM, School for Nutrition and Translation Research Maastricht, Faculty of Health, Medicine and Life Sciences, Maastricht University, Maastricht, The Netherlands; 8 Department of Radiology, University Hospital Nice, Nice, France; 9 Department of Cardiology, University Hospital Nice, Nice, France; 10 Department of Radiology, Centre Hospitalier Universitaire Luxembourg, Luxembourg, Luxembourg; 11 Department of Cardiology, Centre Cardiologique Orban, Liège, Belgium; 12 Department of Cardiology, University Hospitals Leuven, Leuven, Belgium; 13 Department of Cardiovascular Sciences, KU Leuven, Leuven, Belgium; 14 Department of Cardiovascular Sciences, University of Antwerp, Antwerp, Belgium; 15 Department of Cardiology, University Hospital Antwerp, Antwerp, Belgium; 16 Department of Cardiology, Baker Heart and Diabetes Institute, Melbourne, Australia

**Keywords:** Cardiac troponin, T1 mapping, High intensity cycling, Myocardial edema

## Abstract

**Purpose:**

Although cardiac troponin I (cTnI) increase following strenuous exercise has been observed, the development of exercise-induced myocardial edema remains unclear. Cardiac magnetic resonance (CMR) native T1/T2 mapping is sensitive to the pathological increase of myocardial water content. Therefore, we evaluated exercise-induced acute myocardial changes in recreational cyclists by incorporating biomarkers, echocardiography and CMR.

**Methods:**

Nineteen male recreational participants (age: 48 ± 5 years) cycled the ‘L’étape du tour de France” (EDT) 2021’ (175 km, 3600 altimeters). One week before the race, a maximal graded cycling test was conducted to determine individual heart rate (HR) training zones. One day before and 3–6 h post-exercise 3 T CMR and echocardiography were performed to assess myocardial native T1/T2 relaxation times and cardiac function, and blood samples were collected. All participants were asked to cycle 2 h around their anaerobic gas exchange threshold (HR zone 4).

**Results:**

Eighteen participants completed the EDT stage in 537 ± 58 min, including 154 ± 61 min of cycling time in HR zone 4. Post-race right ventricular (RV) dysfunction with reduced strain and increased volumes (*p* < 0.05) and borderline significant left ventricular global longitudinal strain reduction (*p* = 0.05) were observed. Post-exercise cTnI (0.75 ± 5.1 ng/l to 69.9 ± 41.6 ng/l; *p* < 0.001) and T1 relaxation times (1133 ± 48 ms to 1182 ± 46 ms, *p* < 0.001) increased significantly with no significant change in T2 (*p* = 0.474). cTnI release correlated with increase in T1 relaxation time (*p* = 0.002; *r* = 0.703), post-race RV dysfunction (*p* < 0.05; *r* = 0.562) and longer cycling in HR zone 4 (*p* < 0.05; *r* = 0.607).

**Conclusion:**

Strenuous exercise causes early post-race cTnI increase, increased T1 relaxation time and RV dysfunction in recreational cyclists, which showed interdependent correlation. The long-term clinical significance of these changes needs further investigation.

**Trial registration numbers and date:**

NCT 04940650 06/18/2021. NCT 05138003 06/18/2021.

**Supplementary Information:**

The online version contains supplementary material available at 10.1007/s00421-023-05259-4.

## Introduction

It remains unclear if long-duration high-intensity physical exercise in athletes may cause myocardial edema, leading to myocyte necrosis and eventually myocardial fibrosis (La Gerche 2013). Several studies have shown that prolonged endurance exercise is associated with functional and biochemical evidence of myocardial damage with exercise-induced reductions in ventricular strain and concomitant increases in cardiac troponins (cTn) (La Gerche et al. 2012; Klinkenberg et al. 2016). Exercise-induced changes in ventricular function are typically more profound for the right heart, likely due to its greater hemodynamic load during exercise (La Gerche et al. 2011, 2012). Furthermore, exercise-intensity appears to be a more important determinant of reductions in ventricular strain and increases in cardiac troponin than exercise duration (Stewart et al. 2016; Li et al. 2017).

Despite biochemical clues for myocardial injury after high-intensity exercise, the underlying pathophysiology underpinning these changes remains incompletely understood. Potential mechanisms include increased cardiomyocyte membrane permeability, cytotoxic myocardial oedema and cardiomyocyte apoptosis (Aengevaeren et al. 2021a, b). Previous cardiac magnetic resonance (CMR) studies including marathon runners and triathletes were unable to detect myocardial edema following an acute bout of endurance exercise and reported different post-race functional myocardial changes (O'Hanlon et al. 2010) (Mousavi et al. 2009; Tahir et al. 2020). In these studies, myocardial inflammation diagnosis was based on the original Lake Louise criteria (Mousavi et al. 2009; O'Hanlon et al. 2010) or post-exercise CMR was performed too early (~ 2.4 h) after exercise termination (Tahir et al. 2020) missing peak cTn release that is usually observed 3 to 6 h post-exercise (Baker et al. 2019). Coexisting skeletal muscle inflammation may lead to false negative results of CMR using the original Lake Louise criteria, as the T2 ratio represented the signal intensity in the myocardium normalized against reference regions in skeletal muscle (Ferreira et al. 2018). While being an important determinant of exercise-induced cardiac changes (Stewart et al. 2016), exercise intensity and duration have not been considered appropriately in these studies. As such, the exercise intensity in a prolonged duration activity may have been insufficient to induce significant myocardial edema.

New CMR techniques using parametric mapping allow quantification of myocardial T1 and T2 relaxation times with the potential to detect nonischemic myocardial inflammation with a high accuracy compared to older CMR imaging methods (Ferreira et al. 2018; Filomena et al. 2022).

Therefore, the objective of this study was to evaluate the relationship of exercise-induced functional, biochemical, and structural myocardial changes in recreational cyclists, using myocardial native T1 and T2 values as surrogate markers of myocardial edema. We hypothesized that participation in “L’étape du tour de France” (EDT) 2021 (175 km, 3600 altimeters)’ ride would induce significant increase of T1 and/or T2 myocardial relaxation times 3 to 6 h after exercise termination, proportional to the changes in biomarkers and ventricular function.

## Methods

### Study population

This was a prospective observational cohort study approved by the medical ethical committees (Ethische toetsingscommissie Jessa Hospital, Hasselt, Belgium, Comité voor Medische Ethiek, UHasselt, Hasselt, Belgium and Comité de protection des personnes Sud-Ouest et Outre-mer II, Toulouse, France) and all participants gave their written informed consent. Recreational male cyclists performing the EDT 2021 (175 km, 3600 altimeters) ride were eligible for inclusion in this study. Before the race, each participant completed a questionnaire about their medical and training history (hours and kilometers in the last six months). No cyclist participated in any significant endurance event in the preceding week of the race or had regular training sessions within 72 h prior to rest CMR and the EDT 2021 ride. Exclusion criteria for admission were the intake of anti-inflammatory drugs and any systemic or cardiovascular disease.

According to the UCI guidelines, all participants were tested by a polymerase chain reaction (PCR) test within 72 h before the rest examinations to exclude the presence of SARS-CoV-2 RNA.

### Exercise testing

Following cardiac preparticipation evaluation by an experienced cardiologist, all participants performed an exercise test approximately one week before the EDT stage to evaluate their physical fitness. During the exercise test, an electronically braked cycle ergometer (Cylus2^®^, General Electric GmbH, Bitz, Germany) with pulmonary gas exchange analysis (Metalyzer II^®^ 3B Cortex, Leipzig, Germany) was used. The participants started at 100 W during the first 3 min. Hereafter, the workload was increased by 40 W each 3 min and the test was performed until voluntary exhaustion. Participants were asked to cycle at least 70 rpm throughout the test. Oxygen uptake (VO_2_), expiratory volume (VE), and respiratory exchange ratio (RER) were collected breath-by-breath and averaged every minute. At the end of the test, RER values (> 1.1) were evaluated to verify whether the test was performed at full capacity.

Maximal oxygen uptake (VO_2_max) was the primary outcome of exercise capacity. In addition, maximal cycling resistance (*W*_max_), maximal heart rate (HR_max_), and test duration, defined as the corresponding load, heart rate, and minutes at the level of exhaustion, were reported. Recovery heart rate (HR_recovery_) was noted following two minutes of rest after cessation of the exercise test. Capillary blood samples were obtained from the earlobe to analyze blood lactate concentrations (mmol/l). Blood lactate was measured every three minutes, at maximal exhaustion and after recovery (lactate peak) using a portable lactate analyzer (Accutrend Plus, Roche Diagnostics Limited, Sussex, UK).

Heart rate zones were determined using blood lactate concentrations of 2 and 4 mmol/l in combination with RERs of 0.92 and 1, respectively.

### Heart rate monitor and cycling computer data processing

Each participant’s heart rate (HR) zones were individually assigned to the cycling computer device based on the maximal HR, and HR at the aerobic and anaerobic gas exchange thresholds during the exercise test. An HR monitor connected by Bluetooth to the cycling computer was used to ensure the correct registration of HR intensity over time and the ride duration. All participants were asked to cycle 2 h around their anaerobic gas exchange threshold (in HR zone 4) to induce a sufficiently intense exercise stimulus. The HR zones based on the individual exercise testing results gave a more correct and comparable measurement of the exercise intensity of each participant. The race activity files containing HR data and the ride duration were collected after the race.

### HR features

HR data were analyzed for mean and maximal HR values and the intensity profile of each cyclist, including the percentage of total race time spent in HR zone 4. This approach was based on prior studies demonstrating a stepwise cTn increase between mean HR of 140 and 160 bpm and the duration of elevated HR as a predictor of exercise-induced cTn elevation (Stewart et al. 2016; Bjørkavoll-Bergseth et al. 2020).

### Cardiovascular magnetic resonance (CMR)

CMR imaging (3 T Discovery MR 750w, GE Healthcare, Waukesha, WI) was performed one day before and 3–6 h after the race at the same time of the blood sample. Steady-state free precision (SSFP) cine, native T1 and T2 parametric mapping images were obtained. The time point post-exercise was chosen based on the highest troponin release post-exercise allowing enough time for myocardial edema to develop and be detectable, and representing the liberation of enzymes from damaged myocytes (Baker et al. 2019). In addition, a third CMR examination was repeated in a randomly selected subgroup of 8 cyclists approximately 18–20 h post-exercise to document the evolution over time and to confirm the presence of possible subclinical myocardial edema. The imaging parameters are reported in the ‘supplementary material 1’ file.

### CMR postprocessing and analysis

Native T1 and T2 maps were calculated offline pixel-by-pixel from a set of images with different T1- and T2-weighting by two observers (BP and BV) experienced in CMR using in-house Mevislab tool (Vs 2.5.1 MeVis Medical Solutions AG, Bremen, Germany) and commercially available software package (suiteHEART^®^, Version 4.0.6, Neosoft, Pewaukee, Wisconsin, USA). Both readers were blinded to the scan (rest and post-exercise CMR), and clinical and biological data. Maps and error maps were calculated applying a validated retrospective motion correction method of the T1-weighted images (Tilborghs et al. 2019). A freehand region of interest (ROI) was drawn in the septum for global assessment using a fixed color code and range (lut royal; 650–1650 ms (ms) for T1 mapping, 0–80 ms for T2 mapping) and error maps to avoid evident susceptibility artifacts or adjacent blood pool and extra-myocardial tissue pixels (supplemental Fig. 1) (Schulz-Menger et al. 2020).

Cardiac ventricular volumes, function and LV mass were quantified at rest and post-exercise in consensus by two observers using analysis software (suiteHEART^®^, Version 4.0.6, Neosoft, Pewaukee, Wisconsin, USA), according to standardized recommendations (Schulz-Menger et al. 2020). The software automatically defined the endo- and epicardial LV, and endocardial RV contours and was manually adjusted if the tracking was suboptimal. Trabeculae and papillary muscles were included in the LV cavity.

### Two-dimensional transthoracic echocardiography

Two-dimensional transthoracic echocardiography (TTE) was performed using an ultrasound system TUS-AI 900/5L (Aplio i900, Software Version 6.5 Canon medical Systems, Otawara, Japan) with 2D phased array (PST-28BT, Canon medical Systems, Otawara, Japan) transducer. Atrial volumes, and left and right atrial and ventricular deformations were assessed off-line using commercially available software (UltraExtend NX, Software Version 1.0, Canon Medical Systems, Otawara, Japan).

### Biomarkers

All blood samples were collected one day before and at 3–6 h after the stage at the same time of the CMR examination, and analyzed by the Clinical Laboratory, University Hospital Pasteur 2, Nice, France. High-sensitivity cardiac Troponin I levels (cTnI) levels were determined using the cTnI-Ultra assay for the ADVIA Centaur CP (Siemens Healthineers, Erlangen, Germany) with an analytic limit of detection of 6 ng/L. The claimed 10% CV was 30 ng/L was with an upper reference limit of 40 ng/L. C-reactive Protein (CRP) and creatin kinase (CK) concentrations were analyzed using a Roche-Cobas system (Roche Diagnostics GmbH, Mannheim, Germany) with a normal range up to 5 mg/L and < 190 U/L respectively, according to the information provided by the manufacturer.

### Statistical analysis

All statistical analyses were performed by IBM SPSS^®^ version 27.0 (IBM SPSS Statistics for Windows, Chicago, IL, USA). A Shapiro–Wilk test was used to test the normality of the data (*p* < 0.05). All continuous data characteristics were presented as mean ± standard deviation or absolute frequency. Comparisons between baseline and post-exercise values were tested using a paired two-sided Student *t*-test for normally distributed data and a Wilcoxon signed rank test for abnormally distributed data. The Friedman test was used to measure differences in T1 and T2 relaxation times between baseline, 3–6 h post-exercise, and 18–20 h post-exercise within a randomly selected sub-sample of 8 cyclists. Pairwise analyses (Dunn’s post-hoc comparison test) were performed when the Friedman test was statistically significant. A power calculation of our paired t-test was performed based on the standard deviation of a previous study (Tahir et al. 2020) using a significance level of 5% and a sample size of 16, which resulted in a power of 93% for T1 and 98% for T2 relaxation times.

Interobserver reproducibility for T1/T2 mapping measurements was assessed using intraclass correlation coefficient (ICC) estimates and Bland–Altman analysis.

A Pearson correlation was used for normally distributed continuous variables and a Spearman’s rank correlation for not normally distributed data evaluating correlations between post-race troponin changes and the difference between post- and pre-exercise of T1/T2 relaxation time, cardiac function, and volumes, and with cycling intensity parameters. cTnI concentration was not normally distributed and, therefore, we used a natural logarithm to visualize the correlations between post-race cTnI changes, and post-exercise T1 mapping and RVEF modifications. Statistical significance was defined as *p* < 0.05.

## Results

The study population included 19 male cyclists (age 48 ± 5 [range, 42–59] years). Their clinical parameters and cycling performance data are summarized in Table 1. The participant’s exercise testing results were highly variable, reflecting a heterogeneous group of recreational cyclists (Table 2).

**Table 1:** Clinical parameters and training data

*n* = 19 participants	Mean ± SD [range]
*Clinical parameters*	
Age, years	48 ± 5 [42–59]
Weight, kg	79.8 ± 8.5 [64.3–95]
Height, m	1.83 ± 5.6 [1.70–1.91]
Body mass index, kg/m^2^	23.6 ± 2.2 [19.2–27.2]
Heart rate at rest, bpm	60.5 ± 7.0 [48–74]
Systolic BP at rest, mmHg	134.3 ± 9.4 [121–162]
Diastolic BP at rest, mmHg	83.3 ± 4.6 [77–91]
*Training data during 6 months before the EDT stage*	
Hours	123.5 ± 62.6 [36.5–300]
Kilometers	3047 ± 1512 [1072–7400]

Numbers are mean ± standard deviation for continuous data

*EDT* Etape du tour de France, *kg* kilogram, *m* meters, *BP* Blood pressure, *mmHg* millimeters of mercury

**Table 2:** Exercise testing data

*n* = 19 partipants	Mean ± SD [range]
Peak systolic BP, mmHg	170 ± 14 [148–205]
Peak diastolic BP, mmHg	95 ± 14 [73–116]
Heart rate at rest, bpm	75.6 ± 15.1 [50–107]
Peak heart rate, bpm	177.1 ± 9.9 [161–171]
Recovery heart rate, bpm	117.9 ± 15 [94–148]
Aerobic treshold, bpm	142.5 ± 11.9 [117–171]
Anaerobic treshold, bpm	167.1 ± 10.7 [147–186]
Aerobic threshold, watt	202.5 ± 19.1 [173–235]
Anaerobic threshold, watt	285.8 ± 20.2 [260–340]
Maximal power, watt_peak_	350 ± 33 [320–440]
Maximal power, watt_peak(relative)_/kg	4.2 ± 0.6 [3.2–5.4]
VO2_max_, ml/kg per min	53.4 ± 7.3 [44–65]
Duration of the test, min	24.2 ± 2.5 [22–31]
Peak lactate, mmol/l	12.8 ± 2 [8.4–15.6]
Respiratory exchange ratio	1.11 ± 0.03 [1.01–1.15]

Numbers are mean ± standard deviation for continuous data

*BP* Blood pressure, *bpm* beats per minute, *mmHg* millimeters of mercury, *VO2*_*max*_ maximal oxygen uptake, *min* minutes

### SARS-CoV-2 (COVID-19)

Two participants recovered from COVID-19 infection more than one year before the EDT ride without any residual clinical symptoms. At the time of the EDT race, eleven participants were completely vaccinated, seven received one of two doses, and the last received an Ad26.COV2. S vaccine. None of the participants was vaccinated within the week before the EDT stage. All participants were in good health and active COVID-19 infection was excluded by a negative PCR test within 72 before the CMR at rest.

### EDT race data

Eighteen cyclists successfully finished the race in 537 ± 58 min (range 453–635 min).

Battery problems (*n* = 2) and longer periods of missing data (*n* = 2) during the recording of the race activity file by the cycling computer device resulted in an evaluation of cycling intensity in 14 riders. The mean and maximal HR of the 14 cyclists were 131 ± 9 bpm and 167 ± 9 bpm, respectively. Cycling time in HR zone 4 (147 ± 59 min) was highly variable, with minimal and maximal times of 64 and 262 min, representing 29 ± 13% (range 10–47%) of total cycling time.

### Blood sample (Table 3)

**Table 3:** Baseline and post-race serum biomarkers, echocardiography and CMR changes in all cyclists

	Before (*n* = 19)	Post-race (*n* = 18)	*p*-value post vs pre-race
*Blood sample*			
Troponin I, ng/l	1.9 ± 5.1 [0–18.8]	69.9 ± 41.6 [14.4–200.3]	< 0.001
C-reactive protein (CRP), mg/L	1.1 ± 2.1 [0.2–9.5]	4,0 ± 3,1 [0,4–9,6]	< 0.001
Creatin kinase total, ng/L	122.4 ± 52.2 [53–266]	527,2 ± 541,8 [156–2558]	0.006
Creatin kinase-MB, ng/L		26,9 ± 5,8 [21–36]	
*Echocardiography-LV*			
Strain A4C, %	− 19.7 ± 1.7 [− 20.5− (− 18.9)]	− 18.2 ± 1.7 [− 19.0− (− 17.3)]	0.007
GLS, %	− 19.8 ± 1.5 [− 20.5− (− 19.1)]	− 18.8 ± 2.0 [− 19.8− (− 17.7)]	0.047
*Echocardiography-RV*			
Strain RV, FW, %	− 25.5 ± 2.5 [− 26.8− (− 24.3)]	− 23.2 ± 3.2 [− 24.8− (− 21.6)]	0.009
Strain RV A4C, %	− 22.1 ± 2.3 [− 23.2− (− 20.9)]	− 20.5 ± 2.4 [− 21.7− (− 19.4)]	0.025
*Echocardiography-LA*			
LAsr, %	36 ± 13.9 [29.1–42.9]	30.4 ± 10.1 [25.4–35.4]	0.208
LAscd, %	− 22.6 ± 9.3 [− 27.2− (− 17.9)]	− 20.9 ± 9.0 [− 25.4− (− 16.4)]	0.572
LAsct, %	− 13.7 ± 8.6 [-18.0− (− 9.4)]	− 10.1 ± 4.3 [− 12.3− (− 8.0)]	0.204
LA Vol_max_, ml	58.6 ± 17.5 [49.9–67.3)]	55.6 ± 18.4 [46.5–64.7]	0.583
LA Vol_min_, ml	22.4 ± 9.0 [17.8–27.0)]	22.5 ± 10.5 [17.1–27.9)]	0.967
LA Vol_PreA_, ml	38.7 ± 15.4 [30.1–46.6)]	36.8 ± 18.2 [27.5–46.2)]	0.689
LAVI, ml/m^2^	28.6 ± 7.9 [24.7–32.6)]	27.5 ± 9.1 [23.0–32.0)]	0.664
*Echocardiography-RA*			
RAsr, %	41.2 ± 10.5 [35.8–46.6]	34.1 ± 7.8 [30.1–38.1]	0.013
RAscd, %	− 25.7 ± 4.8 [− 28.2− (− 23.3)]	− 19.5 ± 8.0 [− 23.6− (− 15.4)]	0.004
RAsct, %	− 15.5 ± 8.7 [− 20.0− (− 11.0)]	− 14.5 ± 6.4 [− 17.8− (− 11.3)]	0.676
RA Vol, ml	69.6 ± 25.0 [57.2–82]	65.4 ± 19.1 [55.8–75.0]	0.472
RAVI, ml/m^2^	34.4 ± 12.8 [28.1–40.8]	32.1 ± 9.0 [27.6–36.6]	0.426
*CMR-left Heart*			
LV ejection fraction, %	58.4 ± 5.1 [49–68]	57.2 ± 5.8 [45–65]	0.733
LVEDVi, ml/m^2^	100.5 ± 13.3 [81–129]	100.5 ± 15.0 [77.8–130]	0.624
LVESVi, ml/m^2^	42.0 ± 8.5 [28.7–64.4]	43.3 ± 10.7 [31.1–68.2]	0.738
LV cardiac index, l/min/m^2^	3.2 ± 0.6 [2.4–4.5]	4.0 ± 0.7 [3.2–5.6]	< 0.001
LV mass index, g/m^2^	61.8 ± 7.3 [54–85]	62.1 ± 6.2 [54–79]	0.553
*CMR- right Heart*			
RV ejection fraction, %	54.3 ± 4.5 [49–64]	50.8 ± 4.1 [41–61]	0.005
RVEDVi, ml/m^2^	106.7 ± 16.4 [81.1–140]	113.5 ± 15.9 [88.8–142]	0.005
RVESVi, ml/m^2^	48.9 ± 9.7 [29.3–65.1]	56.1 ± 10.8 [36.2–83]	< 0.001
RV cardiac index, l/min/m^2^	3.2 ± 0.6 [2.4–4.6]	4.1 ± 0.7 [3.4–5.6]	< 0.001
Heart rate, beats/min	56 ± 8 [42–71]	67 ± 7 [57–79]	< 0.001
Mapping parameters*			
T1, ms	1133 ± 48 [1045–1251]	1182 ± 46 [1110–1281]	< 0.001
T2, ms	43.9 ± 2.8 [39.2–50.3]	44.1 ± 2.5 [40.1–50.2]	0.474

Numbers are mean ± standard deviation for continuous data. Lower and upper 95% CI between brackets

*CMR* cardiac magnetic resonance, *LA* left atrium, *RA* right atrium, *LV* left ventricle, *A4C* apical 4 chamber, *GLS* the average global longitudinal stain between 2, 3 and 4 apical views, *FW* free wall, *LAsr* left atrial reservoir strain, *LAscd* left atrial conduit strain, *LAsct* left atrial contractile strain, *LA Vol*_*max*_ maximal left atrial volume, *LA Vol*_*min*_ minimal left atrial volume, *LA Vol*_*PreA*_ pre-A-wave left atrial volume, *LAVI* left atrial volume indexed, *RAsr* right atrial reservoir strain, *RASscd* right atrial conduit strain, *RAsct* right atrial contractile strain, *RA Vol* right atrial volume, *RAVI* right atrial volume indexed, *LVEDVi* left ventricular end-diastolic volume index, *LVESVi* left ventricular end-systolic volume index, *RV* right ventricle, *RVEDVi* right ventricular end-diastolic volume index, *RVESVi* right ventricular end-systolic volume index, *ml* millimeter, *ms* milliseconds, *min* minutes

*17 cyclists

**Fig. 1:**
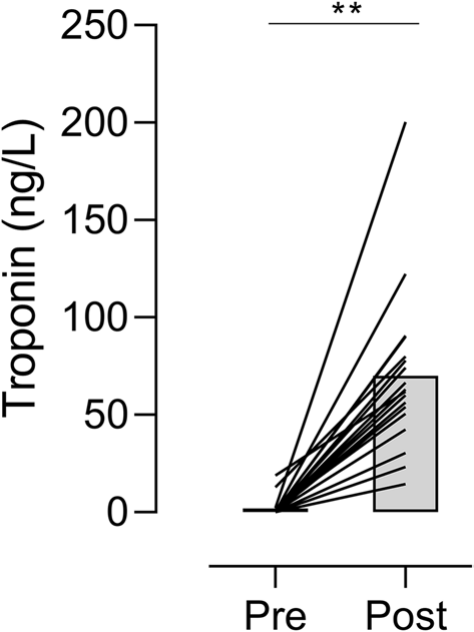
Changes in between pre- and post-exercise** cardiac troponin I levels in 17 cyclists. Data are presented as mean and the individual changes in cardiac troponin I between pre- and post-exercise

The cTnI values at rest were within normal ranges for all participants (1.9 ± 5.1 [0–18.8] ng/l). One cyclist had a CRP level of 9.5 ml/I, without clinical symptoms. cTnI level 3–6 h post-exercise was elevated above the cut-off level for acute myocardial infarction (> 30 ng/L) in 16 of the 18 cyclists (*p* < 0.001) (Fig. 1). Post-race CRP was significantly increased in five cyclists, including the two cyclists without significant cTnI elevation and three cyclists who had fallen during the race.

### Baseline and post-race echocardiographic and CMR findings

Post-race CMR and blood tests were performed 306 ± 63 (range 200–400) minutes after the arrival of the cyclists. No correlations were found between the time difference of CMR/blood sample and post-exercise T1/T2 relaxation time increase or cTnI release (all *p*-values > 0.05).

#### Cardiac volumes and function

Baseline and post-race echocardiographic and CMR measurements are detailed in Table 3. The heart rate was higher at post-exercise CMR than during the rest scan (67 ± 7 bpm versus 56 ± 8 bpm).

**Fig. 2:**
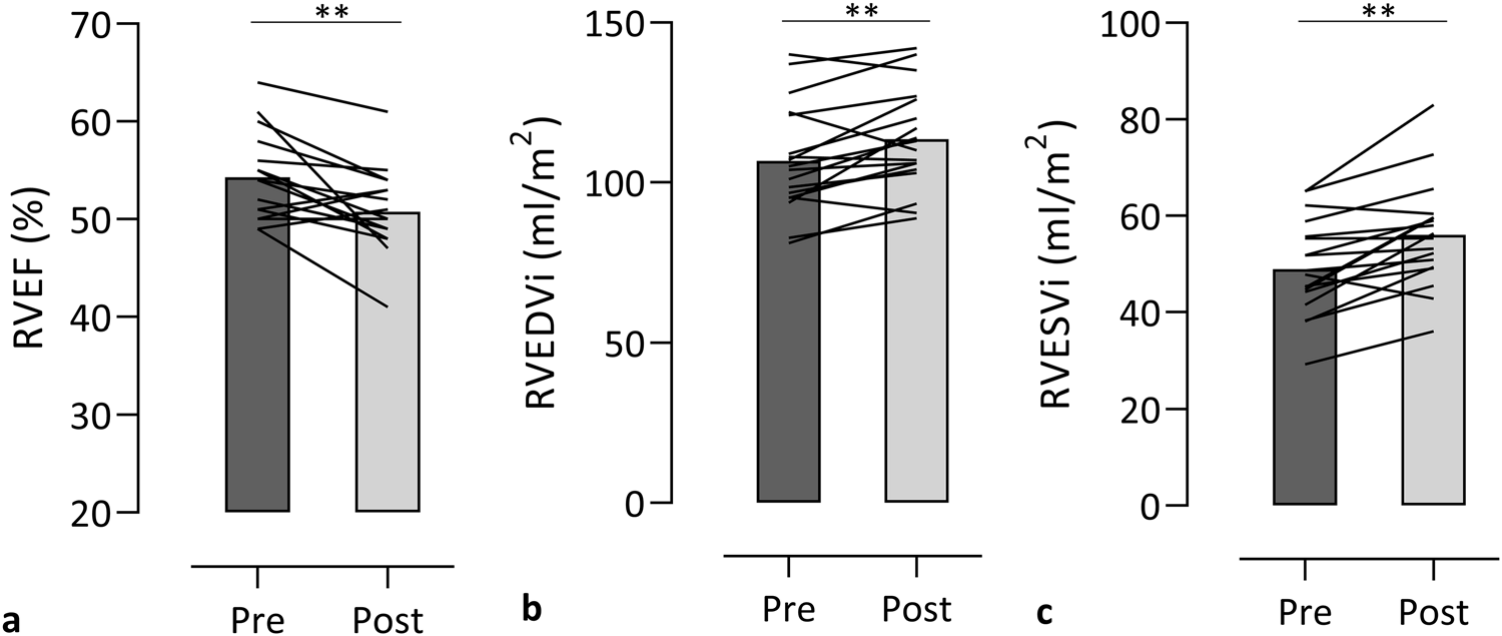
Changes in right ventricular EF (**a**), EDVi (**b**) and ESVi (**c**) between pre- and post-exercise** in 17 cyclists. Data are presented as mean and as the individual changes in right ventricular EF, EDVi and ESVi between pre- and post-exercise. *EF* ejection fraction, *EDVi* end-diastolic volume index, *ESVi* end-systolic volume index, *ml* millimeters

Post-race RVEF was reduced and RV cardiac volumes were increased, while no change in LV function and cardiac volumes was observed on CMR (Fig. 2). On TTE, post-race reductions of strain were more pronounced in the RV than in the LV, with borderline reduced LV global longitudinal strain (GLS). Atrial volumes and left atrial function did not change, while right atrial—reservoir and -conduit strain were significantly reduced following high-intensity cycling.

#### T1/T2 mapping

**Fig. 3:**
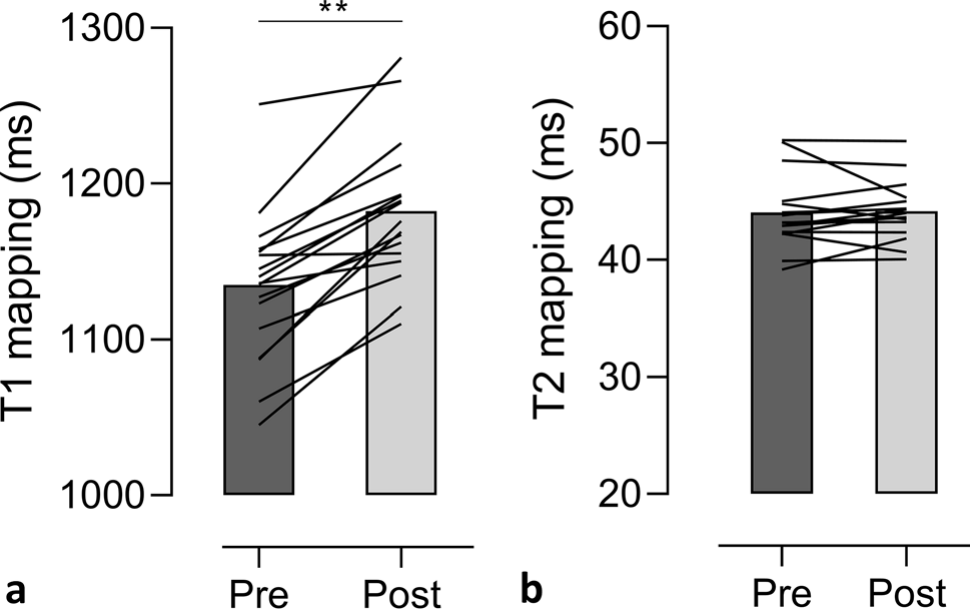
Changes in T1 (**a**) and T2 (**b**) mapping (myocardial T1 and T2 relaxation times) between pre- and post-exercise** in 17 cyclists. Data are presented as mean and the individual changes in T1 and T2 mapping between pre- and post-exercise. *ms* milliseconds

One cyclist was excluded because of non-diagnostic T1/T2 mapping image quality due to ECG-gating problems. Myocardial T1 relaxation times increased significantly post-exercise (1133 ± 48 to 1182 ± 46 ms, *p* < 0.001) in the 17 finishers (Fig. 3a and supplemental Fig. 2), while no significant change in T2 mapping (43.9 ± 2.8 to 44.1 ± 2.5 ms, *p* = 0.474) (Fig. 3b) was observed at SA maps (Table 3). Interobserver reproducibility (ICC, single measures) of blinded native T1 and T2 mapping measurements was excellent: T1: 0.985, 95% confidence interval (CI) 0.972 to 0.992; T2: 0.956, 95% CI 0.919–0.976. Bland–Altman plots with 95% limits of agreement confirmed the close agreement of both observers (supplemental Fig. 3a, b).

A third CMR 18-20 h post-exercise, performed in a randomly selected subgroup of 8 riders, revealed a significant decrease of T1 relaxation times compared to 3–6 h post-exercise (1180 ± 56 ms versus 1130 ± 67 ms, *p* = 0.025) and similar values compared to the rest CMR (1117 ± 50 ms versus 1130 ± 67 ms, *p* = 0.483). T2 relaxation times did not change significantly at 3–6 h (44.8 ± 3.6 ms versus 44.6 ± 3.5 ms, *p* = 0.538) and at 18–20 h (44.8 ± 3.6 ms versus 45.6 ± 4.1 ms, *p* = 0.4) post-exercise compared to rest CMR (all *p*-values > 0.05) in this subgroup.

#### Correlation of exercise-induced Troponin I release with cardiac modifications (post-exercise versus rest)

**Fig. 4:**
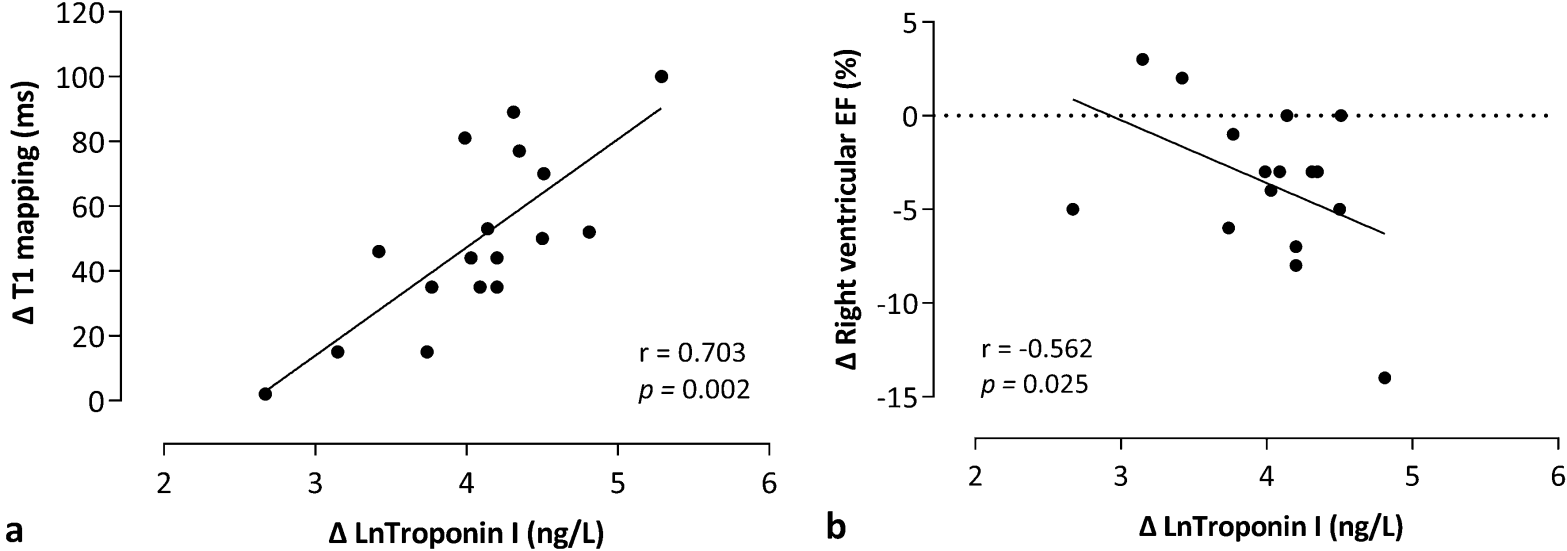
Correlation between exercise-induced Troponin I release and increased post-race T1 myocardial relaxation time (**a**), and right ventricular dysfunction (**b**). Increased post-race T1 myocardial relaxation times (ms) and Troponin I release (ng/L) were strongly correlated (*r* = 0.703; *p* = 0.002) (**a**); more pronounced post-race right ventricular dysfunction was correlated with higher troponin release (*p* = 0.025; *r* = − 0.562) in our cyclists (**b**). Spearman’s rank correlations were used to test correlations between post-race Troponin I changes (Δ LnTroponin I), and post-exercise T1 mapping (Δ T1 mapping) and right ventricular EF (Δ right ventricular ejection fraction) modifications. *ms* milliseconds, *EF* ejection fraction, *Ln* natural logarithm

Post-race cTnI release was strongly correlated with T1 myocardial relaxation time increase (*r* = 0.702, *p* = 0.002) (Fig. 4a). A higher HR difference (post-exercise versus rest CMR) was not related to higher post-exercise T1 myocardial relaxation time (*r* = − 0.235, *p* > 0.05) and troponin modifications (*r* = − 0.385, *p* > 0.05).cTnI release was also correlated with post-race RV dysfunction (*r* = 0.562; *p* = 0.025) in our cyclists (Fig. 4b).

#### Correlation of exercise-induced Troponin I release with cycling intensity

Post-exercise cTnI release was associated with high intensity cycling in HR zone 4 (*r* = 0.607, *p* = 0.03) and inversely with cycling duration (*r* = − 0.514, *p* = 0.03). Faster cyclists rode in HR zone 4 for a higher fraction of total race time (*r* = − 0.656, *p* = 0.01).

## Discussion

Our study demonstrated cTnI release, increased T1 relaxation time and RV dysfunction on CMR following strenuous exercise in recreational cyclists. Moreover, post-race cTnI release correlated with T1 myocardial relaxation time increase, post-exercise RV function reduction and cycling intensity.

### Myocardial edema following strenuous exercise

Post-race cTnI levels were elevated in 16 (89%) of our cyclists, similar to a meta-analysis evaluating biomarker changes after a strenuous endurance exercise (Sedaghat-Hamedani et al. 2015). Moreover, cTnI release was strongly correlated with post-race myocardial T1 relaxation time increase, as a potential marker of subclinical myocardial edema.

In contrast to previous CMR studies, we observed exercise-induced myocardial relaxation T1 increase, which can be explained by several reasons. First, participants performed a much longer (total race time 537 ± 58 versus 198 ± 162 min) and more intensive exercise session (cTnI 70 ± 42 versus 57 ± 86 ng/l) than those from Tahir et al. (2020). Second, the timing of cardiac imaging post-race may have affected the results. The time interval to post-race CMR was longer (306 ± 63 versus 144 ± 162 min) compared to a previous study using parametric mapping in triathletes (Tahir et al. 2020), and based on the time point of the highest cTnI release after the race as extensively documented (Shave et al. 2010; Aengevaeren et al. 2021a, b). Third, pixel-wise parametric mapping was used. This allowed the application of the recently revised 2018 Lake Louise criteria II (Ferreira et al. 2018) to detect myocardial edema with much high accuracy than the original Lake Louise criteria I with T2-weighted CMR imaging used in previous studies (Mousavi et al. 2009; O'Hanlon et al. 2010). Both T1 and T2 mapping can detect subclinical forms of myocarditis. Similar to previous studies, we observed no post-exercise T2 myocardial changes in our cyclists (Aengevaeren et al. 2020). There are several potential explications for this: (1) in contrast to T1 mapping, T2 mapping might be less sensitive to detect a subclinical level of myocardial edema post-exercise; (2) physiological confounders of myocardial T2 mapping, including regional and intersegmental variations and sensitivity to field inhomogeneities and magnetization transfer effects (Wiesmueller et al. 2020); (3) a dehydration status is associated with lower T1/T2 myocardial relaxation times (Luetkens et al. 2020). Although the post-race dehydration of the cyclists was not measured, a more hydrated status compared to rest CMR is almost impossible after such a strenuous and prolonged exercise. A higher HR was associated with lower *T*_2_ values in a previous study (von Knobelsdorff-Brenkenhoff et al. 2013), and our study did not show a correlation between post-exercise HR and T1 increase. Although a pathological T1 and T2 mapping will increase specificity for diagnosing acute myocardial edema, having only a T1 mapping-based marker still support a diagnosis of acute myocardial edema with an area under the curve (AUC) of 89% (Ferreira et al. 2018). A recent meta-analysis to detect acute myocarditis confirmed a higher AUC of native T1 mapping compared to T2 mapping (0.95 versus 0.88) (Kotanidis et al. 2018). The mean T1 relaxation times at rest (1133 ± 48 ms) in our study were concord with the 95% tolerance interval of previous 3 T studies in this age category (von Knobelsdorff-Brenkenhoff et al. 2013; Roy et al. 2017).

The association between cardiac troponin levels and acute myocardial necrosis after endurance sports remains speculative. Post-race cardiac troponin elevations usually return to normal within 24 h suggesting no associated myocardial necrosis. A third CMR scan 18–20 h post-EDT race showed normalization of myocardial T1 relaxation time in a subgroup of 8 cyclists suggesting that just as reported for exercise-induced cardiac troponin changes, these alterations are transient (Aengevaeren et al. 2021a, b). An “increased cardiomyocyte membrane permeability” has been proposed as the major cause of post-exercise cTn release (Sedaghat-Hamedani et al. 2015). However, although there is no direct evidence of post-exercise cytotoxic myocardial oedema, a small degree of myocardial necrosis cannot be completely excluded because of post-exercise cTn elevations (Aengevaeren et al. 2021a, b).

### Myocardial function after endurance exercise

The use of multiple imaging modalities in this study strengthened our findings. CMR showed reduced post-race RV function and increased volumes, while echocardiography demonstrated more pronounced post-race strain reductions of the right heart compared to the left heart. LV was less affected post-race with no significant post-race changes in LV function and cardiac volumes, as previously reported on TTE (La Gerche et al. 2012; Stewart et al. 2017). Exercise-induced RV functional limitations were more profound than LV, likely due to disproportionate RV wall stress during exercise (La Gerche et al. 2015). In contrast to other CMR studies (O'Hanlon et al. 2010; Aengevaeren et al. 2020; Tahir et al. 2020), the post-exercise RV impact on our cyclists can be explained by the higher intensity and longer duration of the exercise activity.

### High-intensity long-duration exercise and clinical implications

High intensity exercise in a prolonged duration cycling activity resulted in more post-race cTn release in our participants, as previously reported (Kleiven et al. 2019; Bjørkavoll-Bergseth et al. 2020; Aengevaeren et al. 2021a, b), while exercise-induced T1 myocardial modifications on CMR were transient, similar to cardiac troponin changes (Aengevaeren et al. 2021a, b). Myocardial inflammation and necrosis following strenuous exercise were worsened during viral infection in animal studies (Ilbäck et al. 1989). Therefore, our study adds to the body of evidence that it is important to avoid high-intensity exercise during (viral) infections. Exercise-induced subclinical edema may be an underlying mechanism of acute post-race RV dysfunction in recreational cyclists. Although the thin RV free wall prevents measurements of subclinical oedema with current parametric mapping techniques due to insufficient spatial resolution (Messroghli et al. 2017), it is a likely that oedema is also present in the RV wall given its expected diffuse nature and the greater hemodynamic impact of exercise on the RV. Biventricular interaction may be an additional mechanism to explain the observed reduction of RV function (La Gerche and Claessen 2019). A further prospective investigation is required to evaluate the recovery time for acute myocardial injury (ea. cTn-related T1 myocardial modifications and RV dysfunction) following (repeated) high-intensity cycling (De Bosscher et al. 2022; Eijsvogels Thijs and Aengevaeren Vincent 2022). An important shortcoming of previous studies is that only a single post-exercise assessment of associations between biochemical, functional, and structural myocardial changes has been evaluated. Detailed information about the recovery time for transient myocardial injury is lacking. Further imaging studies should address more precisely post-exercise myocardial T1/T2 kinetics and functional modifications, and their association with post-race cTn release and with exercise intensity and duration.

### Limitations

There are limitations to our study, including the relatively small sample size (*n* = 19), like many studies of this type. Also, due to above-mentioned technical problems a limited number of 14 files were available for HR zone measurements. The measurement of cycling time in HR zone 4 as a parameter of high intensity, was based on commercially available cycling computers and HR monitor devices, similar to previous studies (Bjørkavoll-Bergseth et al. 2020). The relationship between T1/T2 mapping and exercise parameters was not fully evaluated in this study due to undersampling of our study population and to the high variability of race intensity and duration. No late gadolinium enhancement and extracellular volume calculation were performed to exclude focal myocardial fibrosis. However, this was not the objective of our study and abnormal native T1/T2 mapping provide evidence for acute myocardial edema according to the updated 2018 Lake Louis Criteria (Ferreira et al. 2018). Hydration status was not measured in this study. Its effect on T1/T2 relaxation times could therefore not be assessed, although post-exercise dehydration might have contributed to reduced myocardial relaxations times (Luetkens et al. 2020). Finally, myocardial diffusion-weighted imaging to evaluate myocardial edema was not available on the MR system. Apparent diffusion coefficient maps showed improvement in the depiction of myocardial edema compared to T1/T2 mapping in a recent study (Moulin et al. 2020).

In conclusion, strenuous exercise causes cTnI increase, prolongation of myocardial T1 relaxation time and RV dysfunction in recreational cyclists, which showed interdependent correlation. Exercise-induced myocardial T1 increase may represent subclinical myocardial edema. More research is needed to determine the clinical significance (normal physiological changes or precursors to long-term remodeling) of these acute functional and structural myocardial changes following repeated high-intensity cycling.

## Supplementary Information

Below is the link to the electronic supplementary material.

Supplementary file1 (DOCX 20 KB)

Supplementary file2 (TIFF 7170 KB)

Supplementary file3 (TIFF 33989 KB)

Supplementary file4 (TIFF 337 KB)

## Data Availability

The data generated and analysed during the current study are available from the corresponding author on reasonable request.

## Abbreviations

AUC: Area under the curve
BPM: Beats per minute
CMR: Cardiac magnetic resonance
CRP: C-reactive protein
CTn: Cardiac troponin
EDT: L’étape du tour de France
GLS: Global longitudinal strain
HR: Heart rate
ICC: Intraclass correlation coefficient
MSEC: Milliseconds
PCR: Polymerase chain reaction
LV: Left ventricle
RV: Right ventricle
TTE: Transthoracic echocardiography
