# Supplementary material for: Exercise-induced myocardial T1 increase and right ventricular dysfunction in recreational cyclists: a CMR study
Source: Eur J Appl Physiol. 2023 Jul 22;123(10):2107–17. doi: 10.1007/s00421-023-05259-4 (PMC10492712; doi:10.1007/s00421-023-05259-4)
Supplement: Supplementary file 1 — Supplementary file1 (DOCX 20 KB) [file 421_2023_5259_MOESM1_ESM.docx]

**Exercise-induced myocardial T1 increase and right ventricular dysfunction in recreational cyclists: a CMR study**

**European journal of Applied Physiology**

**Authors:**

Olivier Ghekiere MD PhD^1,2^, Lieven Herbots MD PhD^2,3^, Benjamin Peters MD^1,2^, Baptiste Vande Berg MD^4^, Tom Dresselaers PhD^4^, Wouter Franssen PhD^5,6,7^, Bernard Padovani MD PhD^8^, Dorothee Ducreux MD^8^, Emile Ferrari MD, PhD^9^, Alain Nchimi, MD PhD^10^, Sophie Demanez MD^11^, Ruben De Bosscher MD^12^, Rik Willems MD PhD^12,13^, Hein Heidbuchel, MD PhD^14,15^, Andre La Gerche MD PhD^16^, Guido Claessen MD PhD^2,3*^, Jan Bogaert MD PhD^4^ *, Bert O. Eijnde PhD^5^*

*Shared last author

**Affiliations:**

1. Department of Radiology and department of Jessa & Science/LCRC (-MHU), Jessa Hospital, Hasselt, Belgium
2. Faculty of Medicine and Life Sciences/ LCRC (-MHU), Hasselt University, Diepenbeek, Belgium
3. Department of Cardiology, Heart Centre, Jessa Hospital, Hasselt, Belgium
4. Department of Radiology, University Hospitals Leuven, Leuven, Belgium
5. SMRC Sports Medical Research Center, BIOMED Biomedical Research Institute, Faculty of Medicine and Life Sciences, Hasselt University, Diepenbeek, Belgium
6. REVAL-Rehabilitation Research Center, Faculty of Rehabilitation Sciences, Hasselt University, Diepenbeek, Belgium
7. Department of nutrition and Movement Sciences; NUTRIM, School for Nutrition and Translation Research Maastricht, Faculty of Health, Medicine and Life Sciences, Maastricht University, Maastricht, The Netherlands
8. Department of Radiology, University Hospital Nice, Nice, France
9. Department of Cardiology, University Hospital Nice, Nice, France
10. Department of Radiology, Centre Hospitalier Universitaire Luxembourg, Luxembourg
11. Department of Cardiology, Centre Cardiologique Orban, Liège, Belgium
12. Department of Cardiology, University Hospitals Leuven, Leuven, Belgium
13. Department of Cardiovascular Sciences, KU Leuven, Leuven, Belgium
14. Department of Cardiovascular Sciences, University of Antwerp, Antwerp, Belgium
15. Department of Cardiology, University Hospital Antwerp, Antwerp, Belgium
16. Department of Cardiology, Baker Heart and Diabetes Institute, Melbourne, Australia

**Corresponding author:**

Olivier Ghekiere

Department of Radiology

Jessa Hospital Hasselt

Stadsomvaart 11

3500 Hasselt, Belgium

E-mail: [olivier.ghekiere@jessazh.be](mailto:olivier.ghekiere@jessazh.be)

**Methods**

**Cardiovascular Magnetic Resonance (CMR)**

CMR imaging was performed using a 16-channel phased array torso receive coil, posterior elements embedded in the scanner table and ECG for cardiac gating

Cine imaging

To assess left and right ventricular function, volume and mass, steady-state free precision (SSFP) cine-images were acquired in the short-axis (SA) plane, and in the long axis two-chamber (2CH), three-chamber (3CH) and four-chamber (4CH) views. Imaging parameters were: repetition time (TR) 3.6 ms; echo time (TE) 1.4 ms, phase FOV = 0.90, flip angle (FA) 50°, field of view (FOV) (340 x 340) mm^2^, matrix 160 x 224, slice thickness 8 mm, gap 2 mm, receiver bandwidth (BW) 125 Hz/px, parallel imaging using 30 reconstruction, 30 cardiac phases.

T1 mapping

Native T1 mapping images were acquired using a 5s(3s)3s modified look-locker inversion recovery (MOLLI) sequence at end-diastole within one expiration breath-hold in a short axis (SA) plane. Imaging parameters were: TR= 3.4 ms, TE=1.5 ms, flip angle= 35°, FOV= 360 x 324 mm^2^, matrix 160 x 180 mm, slice thickness = 8 mm, BW = 100 Hz/px, SENSE/acceleration factor = 2.

T2 mapping

For T2 parametric mapping, images were obtained using a fast spin-echo bright blood sequence with 4 sources images at end-diastole within one expiration breath-hold in the same SA plane corresponding to the T1 mapping MOLLI sections. Specific imaging parameters were: variable TR according to the heart rate, TE 11.6 ms, flip angle = 90°, FOV 360 x 324 mm^2^, matrix 180 x 180 mm, slice thickness= 8mm, BW = 83.33 Hz/px, acceleration factor = 1,5.

**Supplemental figures**

**Figure 1 Analysis of T1 and T2 relaxation time on short-axis T1 and T2 mapping maps**

T1 and T2 myocardial relaxation times were calculated by drawing freehand regional region of (C) using a fixed color code and range (lut royal; 650-1650 ms for T1 mapping and 0-80 ms for T2 mapping). Corresponding error maps (Figure 1B and 1D) were used to avoid evident susceptibility artifacts or adjacent blood pool and extra-myocardial tissue pixels.

ms = milliseconds

**Figure 2. Changes of myocardial T1 relaxation time on short axis T1 colormaps at post-exercise versus rest CMR in 17 cyclists**

Note the more orange and red color of the native T1 mapping at 3-6h post-exercise CMR compared to the rest CMR scan in almost all cyclists, related to the increased post-exercise T1 myocardial relaxation time. Scale T1 myocardial relaxation times: 650-1650 ms. CMR = Cardiac Magnetic Resonance Imaging; ms = milliseconds

**Figure 3. 95% Confidence interval Bland–Altman plot of the mean difference (ms) between both observers versus all T1 mapping (a) and T2 mapping (b) measurements**

ms = milliseconds
