# Supplementary figures and images for: Exercise-induced myocardial T1 increase and right ventricular dysfunction in recreational cyclists: a CMR study
Source: Eur J Appl Physiol. 2023 Jul 22;123(10):2107–17. doi: 10.1007/s00421-023-05259-4 (PMC10492712; doi:10.1007/s00421-023-05259-4)

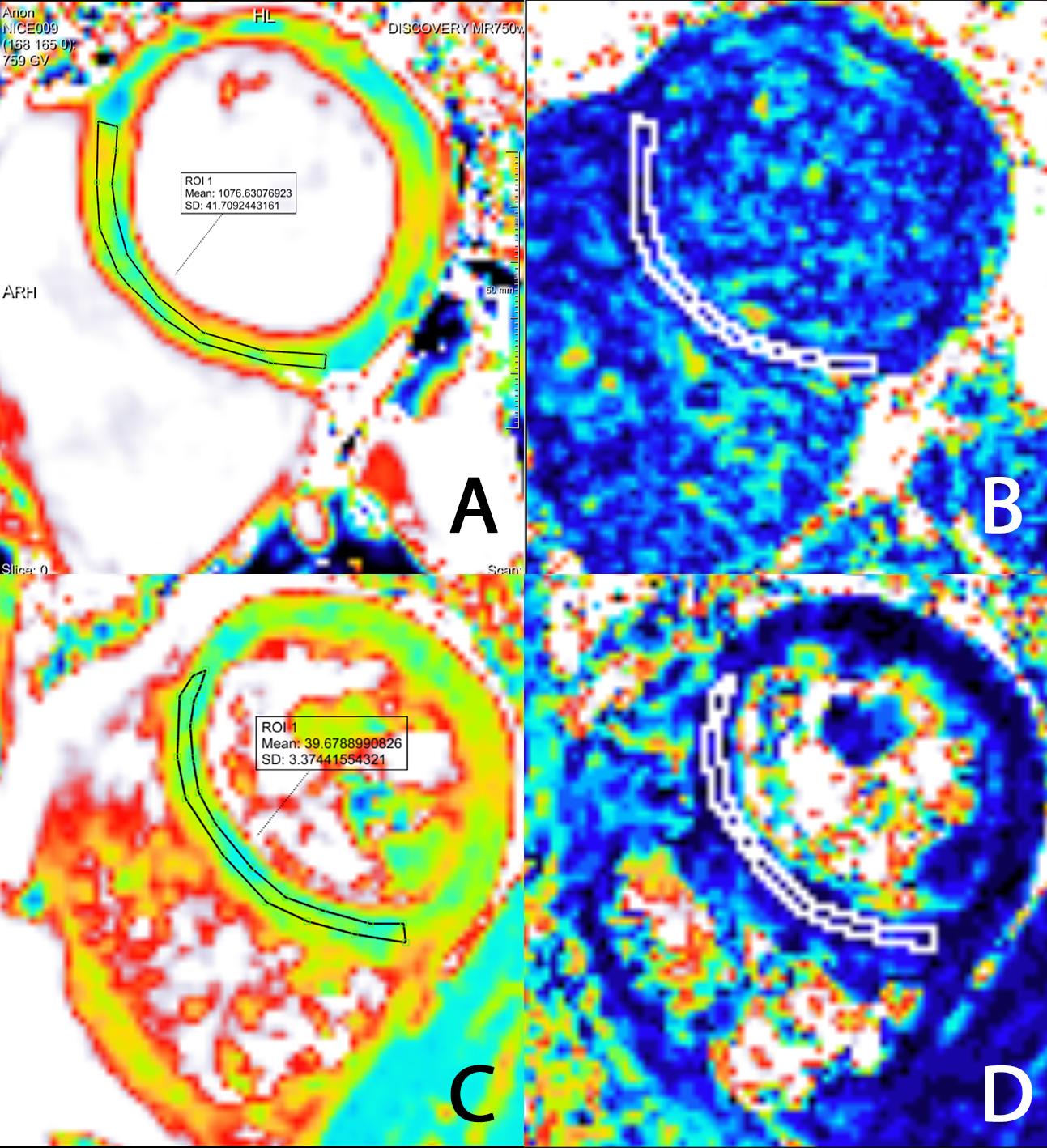

Supplement: Supplementary file 2 — Supplementary file2 (TIFF 7170 KB) [file 421_2023_5259_MOESM2_ESM.tiff]

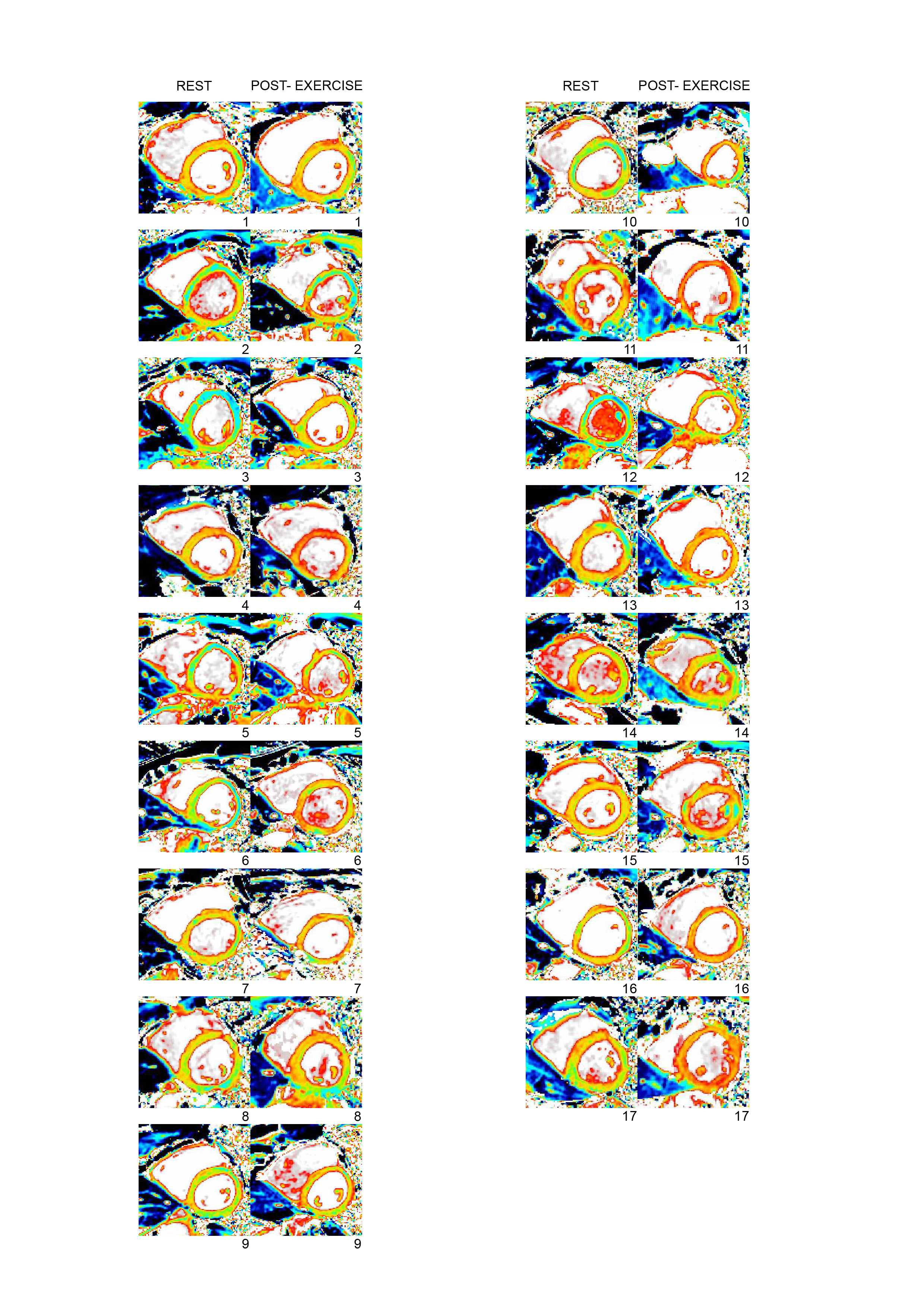

Supplement: Supplementary file 3 — Supplementary file3 (TIFF 33989 KB) [file 421_2023_5259_MOESM3_ESM.tiff]

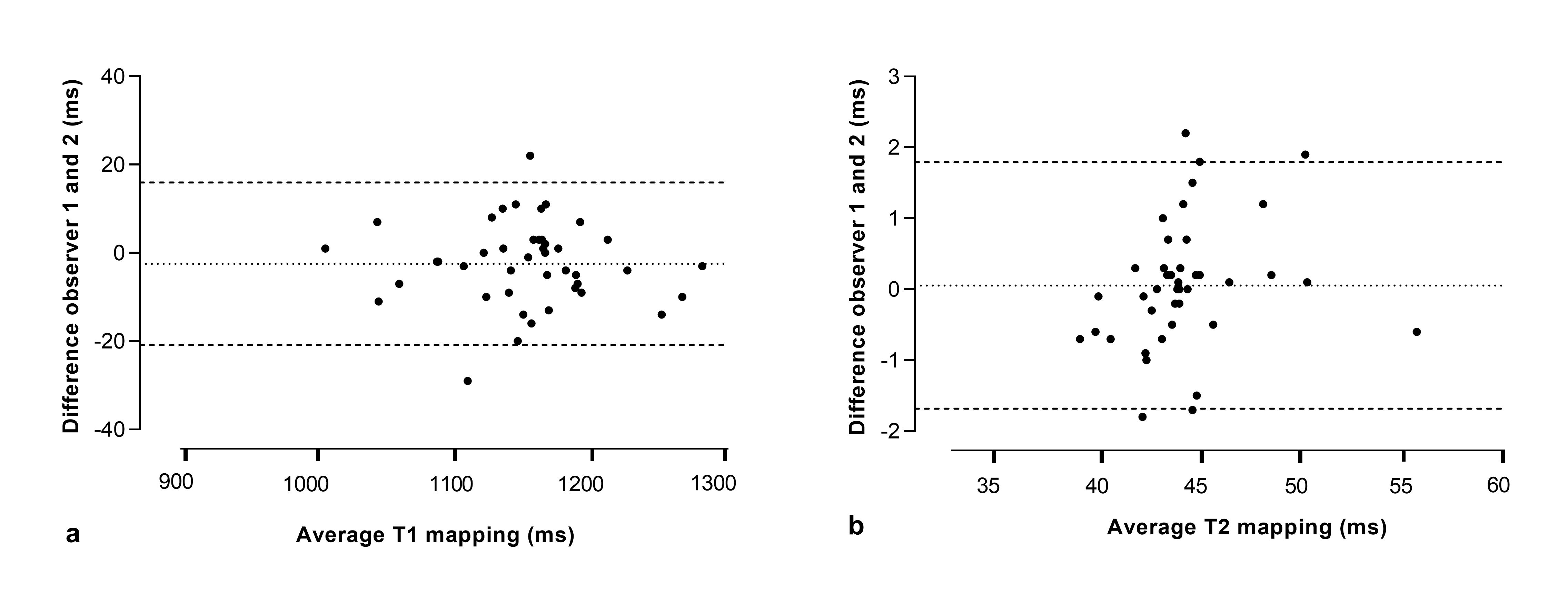

Supplement: Supplementary file 4 — Supplementary file4 (TIFF 337 KB) [file 421_2023_5259_MOESM4_ESM.tiff]
